# Trajectory analyses of virologic outcomes reflecting community-based HIV treatment in Washington DC 1994–2012

**DOI:** 10.1186/s12889-015-2653-x

**Published:** 2015-12-22

**Authors:** Joanne Michelle F. Ocampo, Michael Plankey, Kai Zou, Jeff Collmann, Cuiwei Wang, Mary A. Young, Chenglong Liu, Joshua A. Ripple, Seble Kassaye

**Affiliations:** The Office of the Senior Vice President for Research, Georgetown University, Washington, DC USA; Department of Microbiology and Immunology, Georgetown University Medical Center, Washington, DC USA; The Women’s Interagency HIV Study, Georgetown University Medical Center, Washington, DC USA

**Keywords:** HIV, Viral suppression, Trajectory analysis, HIV treatment career

## Abstract

**Background:**

Effective treatment of HIV since 1996 has reduced morbidity and mortality through virologic suppression. Combination antiretroviral therapy (cART) has been recognized as key to the prevention of drug resistance and the transmission of infection. We used eighteen years of virologic outcomes in a long-standing cohort of women to describe longitudinal viral load trajectories; and examine factors associated with sustained viremia and mortality.

**Methods:**

We analyzed data from DC WIHS women with > four semiannual visits using a group-based logistic trajectory analysis approach to identify patterns of HIV RNA detection (>80 copies/mL or lower assay limit, and >1000 copies/mL). We verified findings using cumulative viral load suppression-years, explored group characteristics using generalized linear modeling with generalized estimating equations for repeated measures, and examined survival using the Kaplan-Meier and Cox proportional hazard analyses.

**Results:**

329 women contributed 6633 visits between 1994 and 2012 and demonstrated high, moderate, and low probability patterns of HIV RNA detection (>80 copies/mL) in 40.7, 35.6, and 23.7 % of participant visits, respectively. Analysis of cumulative years of viral load suppression supported these observations. Kaplan-Meier survival analysis demonstrated high mortality of 31.1 % with sustained viremia, but no significant difference in mortality between intermittent viremia and non-viremia patterns, 6.9 and 4.9 % respectively. Mortality was associated with higher age, lower CD4+ T lymphocyte count, and sustained viremia by Cox multivariate analysis.

**Conclusions:**

This ecologic study demonstrates the effectiveness of viral suppression, and conversely the association between viremia and mortality. In community delivery of cART for HIV care, distinct patterns of sustained viremia, intermittent viremia, and non-viremia were identified over nearly 18 years in the DC WIHS, capturing the dynamics and complexity of sustaining long-term HIV care. Persistent viremia was associated with lower CD4s and mortality, but surprisingly mortality was not different between continuous suppression and intermittent viremia. Classification of long-term virologic patterns such as these observed HIV treatment “careers” may provide a suitable framework to identify modifiable factors associated with treatment resilience and failure. Both individual and population interventions are needed to reduce transmission, prevent the emergence of drug resistance, and improve outcomes of community ART programs.

## Background

Optimal HIV management in the contemporary era of potent and well-tolerated regimens includes early engagement in care and initiation of combination antiretroviral therapy (ART) to achieve viral suppression, promote immune recovery to decrease HIV-associated morbidity and mortality, and decrease risk of HIV transmission [1–3]. HIV RNA testing provides a measure of treatment success and is used as a patient management tool. The HIV care continuum provides a population-level assessment of engagement and retention in care, with the desired ultimate goal to achieve viral suppression [4–9]. However, the current HIV care continuum model does not capture the long-term care perspective of the individual’s progress towards achieving and maintaining viral suppression.

Identification of facilitators and barriers to the achievement and maintenance of viral suppression requires an understanding of long-term individual-level HIV care and treatment dynamics. The concept of an “illness career” which has been successfully applied to other chronic diseases such as mental illness, enables identification of a wide-range of health-seeking behaviors, and outcomes in the context of evolving treatment guidelines [10–12]. For example, the shifting care environment surrounding individuals living with HIV has implications on the interaction between that person and their treatment infrastructure, its overall impact on individual treatment success, and ultimately on controlling the HIV/AIDS epidemic [13].

As with any chronic illness it is important to account for gaps in the care continuum due to structural, biographical, and clinical factors. Thus, understanding the influences that determine the type of illness career, or as proposed here; HIV treatment career (dynamic developmental trajectories that people living with HIV experience throughout a lifetime) for individuals living with HIV is crucial to improve health outcomes and mitigate ongoing HIV transmission. Although trajectory analyses have been applied to other areas within HIV/AIDS including examining longitudinal patterns of stimulant drug use, such methods have not yet been applied to understanding the longitudinal HIV care continuum [14–16]. Here, we 1) describe longitudinal viral load trajectories; 2) examine factors associated with long-term sustained viremia, and; 3) investigate the relationship between long-term sustained viremia and mortality in the Metropolitan District of Columbia, Women’s Interagency HIV Study (DC WIHS) from 1994 to 2012.

## Methods

### Study population

Data for this study was obtained from the Washington DC Metropolitan WIHS site which is housed at Georgetown University in Washington DC with sub-sites in Montgomery County Maryland and in northern Virginia. WIHS is an ongoing prospective cohort study of HIV infection in women. WIHS recruited women from six sites (Bronx and Brooklyn, New York; Chicago, Illinois; Los Angeles and San Francisco, California; and, Washington, DC) during 3 phases (1994–1995; 2000–2001; 2012–2013) [17]. Data from all three waves of the Washington DC site were used for this analysis. Details of recruitment and enrollment for WIHS have been described previously [17, 18]. The DC WIHS recruited women through community outreach and among care providers within DC, and is not a clinic-based cohort. Within this non-intervention observational cohort, health outcomes reflect the local treatment practices and health-seeking behaviors of the participants [17, 18]. For this study, DC WIHS HIV-positive participants who contributed at least four visits over the course of the study were included in this analysis.

### Outcomes

Laboratory collection methods and measurements of viral load (plasma HIV-1 RNA) and CD4+ cell count included isothermal nucleic acid sequence-based amplification and standard flow cytometric protocols, respectively, and have been previously described.[19] HIV RNA detection levels over time were set at the level below assay detection (which varied from <80 copies/mL in semi-annual visits 1–28; <48 copies/mL in visits 29–33; and, < 20 copies/mL for visits 34–36).

Viral load suppression for an individual at a particular visit was defined as viral load less than or equal to the detection limit at the time of the assay. For each visit for which there were data, individuals were assigned 1 for suppression variable if viral suppression was achieved, or 0 if not. Cumulative viral load suppression-year was defined for each individual at each visit by summing suppression values for the current and all prior visits and dividing by two.

Time of report of death during study for any cause was used as the mortality outcome. Ascertainment and classification of deaths in WIHS have been previously described [20, 21].

### Covariates

Demographic variables were recorded with survey questionnaires. Covariates from this questionnaire include the constants race (black defined as non-Hispanic black, Hispanic defined as Hispanic of any race, other defined as non-white, non-black, non-Hispanic, and, referent of white defined as non-Hispanic white) and education (> = 12 years of school with <12 years as referent), and the time-varying variables housing (reporting having own home or, apartment with the following included in the referent of non-housed: living in a parent’s house; someone else’s house, or apartment; a rooming, boarding, or halfway house; a shelter or welfare hotel; the street; jail or correctional facility; a residential drug or alcohol treatment facility; other place; or no report), depressive symptoms (Center for Epidemiologic Studies Depression Scale; CES-D > =16 at visit with CES-D < 16 as no depressive symptoms referent), illicit drug abuse (reported use of at least one of the following since the last visit: marijuana or hash; crack; cocaine; heroine; illicit methadone; methamphetamines; amphetamines, narcotics, hallucinogens, and other drugs; injected drugs; or non-injected drugs. Alcohol was not included), and alcohol use (use defined as > = 7 reported drinks per week since last visit).[22] HIV medication use was also reported with a questionnaire using time-varying variables adherence to treatment (taking HIV drugs > =95 % of the time with <95 % use as referent) and therapy (ART, including mono therapy or combination therapy; cART, including at least three antiretrovirals from at least two drug classes based on the Department of Health and Human Services 2008 guidelines;[23] and no ART/cART treatment as referent). Age (in years) was used as constant (age at participant’s baseline visit in study) or time-varying (age at visit) depending on the analysis.

### Statistical methods

Descriptive statistics using data from the baseline visit were generated. Group-based trajectories were modeled using a logistic trajectory model as a function of visits (PROC TRAJ, available online: http://www.andrew.cmu.edu/user/bjones/) with HIV RNA detection as a binary variable. The optimal number of trajectories was selected based on the Bayesian information criteria (BIC); the model with the lowest BIC value representing the statistically optimal number of latent groups. Group characteristics were explored with generalized linear modeling with generalized estimating equations for repeated measures using PROC GENMOD. Variables from univariate analyses with *P* < 0.1 were included in multivariate models. The overall lost to follow up rate in this specific study group was calculated using the number of individuals classified as “missing” and “disenrolled” in the DC WIHS. Overall mortality in this specific study group was calculated using the number of deceased recorded by DC WIHS. Median viral load and the interquartile range were calculated using viral load reports over time in the study group.

A graph depicting cross sectional proportion of DC WIHS women with viremia and those without viremia was plotted to compare HIV care continuum with results from longitudinal group-based trajectory analyses. Data used included only observations with recorded values and excluded deaths and missing data.

Mean value of viral suppression cumulative years of each visit for each of the three HIV treatment careers was calculated and plotted. To probe the relationship between HIV treatment careers and structural, biographical and clinical factors, both univariate and multivariate multinomial logistic regression analyses were conducted. For time-varying predictors, random effects for subjects were included to account for repeated measures.

Kaplan-Meier survival analysis was performed to identify differences in mortality between the trajectory groups, and univariate and multivariate Cox proportional hazards modeling was conducted to identify predictors of survival. For Cox regression, race, age at entry to study, education, and group variables were treated as constant whereas remaining predictors were treated as time varying.

All analyses were performed in SAS 9.4 64-bit and statistical significance was defined as *P* <0.05.

### Role of the funding sources

The National Institute of Allergy and Infectious Diseases (NIAID) (UO1-AI-34994; PI: Mary A. Young) and the National Cancer Institute, the National Institute on Drug Abuse and the Eunice Kennedy Shriver National Institute of Child Health and Human Development funded the collection of data for this study from the Washington DC Metropolitan site of the Women’s Interagency HIV Study (WIHS), and the Office of the Senior Vice President for Research at Georgetown University funded additional analytical support.

## Results

**Fig. 1:**
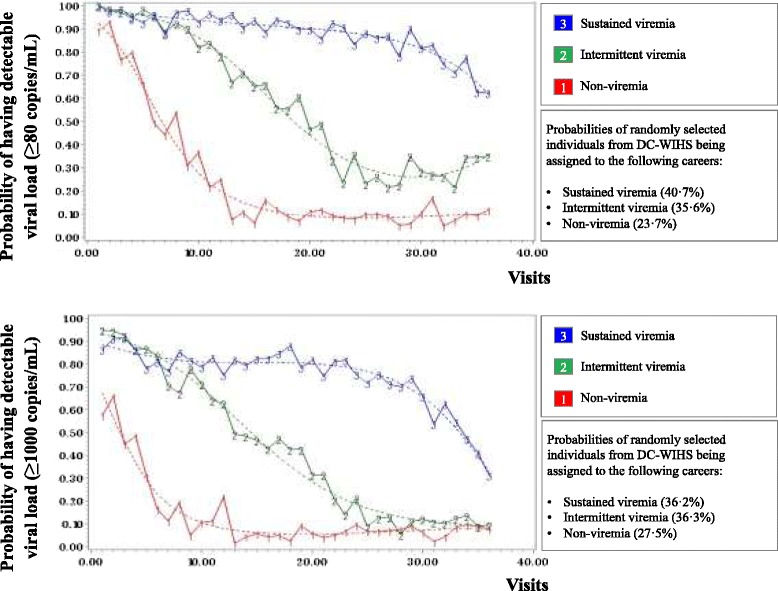
**a** Group-based trajectory analysis with HIV RNA detection level >80 copies/mL or lower detection limit at time of assay from 329 DC WIHS women contributing 6633 participant visits illustrate three distinct HIV treatment careers. **b** Group-based trajectory analysis with HIV RNA detection level >1000 copies/mL from 329 DC WIHS women contributing 6633 participant visits illustrate three distinct HIV treatment careers

In the DC WIHS, 329 women with a median age of 35 years at study enrollment (Table 1) contributed 6633 visits at six-month intervals between 1994 and 2012 and demonstrated three HIV treatment careers with high (sustained viremia), moderate (intermittent viremia), and low (non-viremia) probabilities for having detectable HIV RNA >80/48/20 copies/mL based on the assay detection limit in 40.7, 35.6, and 23.7 % of participant-visits, respectively (Fig. 1a), and HIV RNA >1000 copies/mL in 27.5, 36.3, and 36.2 % of participant-visits, respectively (Fig. 1b). In comparison, the cross-sectional analysis conducted to depict the proportion of individuals with viral suppression from 1994 to 2012 over five-year intervals demonstrated that approximately 60 % of individuals would be defined as having achieved viral suppression over the last 2.5-year period of the study (Fig. 4). Overall lost to follow up and mortality rates in this study group were 18 and 24 %, respectively. Median viral load and (interquartile range) within the trajectories were: sustained viremia 11,250 (2500–36,500), intermittent viremia 110 (80–980), and non-viremia 80 (80–80).

**Table 1:** Descriptive data for study population in DC WIHS

Numeric (mean and standard deviation) and categorical (count and percentage) variables	All Participants at study entry (*N* = 329)	Non-viremia individuals at study entry (*N* = 80)	Intermittent viremia individuals at study entry (*N* = 95)	Sustained viremia individuals at study entry (*N* = 154)
Race				
White	48 (14 · 6 %)	16 (20 %)	16 (16 · 8 %)	16 (10 · 4 %)
Black	245 (74 · 5 %)	47 (58 · 8 %)	69 (72 · 6 %)	129 (83 · 8 %)
Hispanic	26 (7 · 9 %)	14 (17 · 5 %)	7 (7 · 4 %)	5 (3 · 3 %)
Other	10 (3 %)	3 (3 · 8 %)	3 (3 · 2 %)	4 (2 · 6 %)
Education				
< 12 years	97 (29 · 5 %)	15 (18 · 8 %)	26 (27 · 4 %)	56 (36 · 4 %)
> = 12 years	232 (70 · 5 %)	65 (81 · 3 %)	69 (72 · 6 %)	98 (63 · 6 %)
Age	34 · 92 (7 · 6)	34 · 59 (6 · 7)	34 · 15 (7 · 8)	35 · 57 (7 · 9)
Housing				
No housing	102 (31 %)	19 (23 · 8 %)	32 (33 · 7 %)	51 (33 · 1 %)
Housing	227 (69 %)	61 (76 · 3 %)	63 (66 · 3 %)	103 (66 · 9 %)
Depression				
Depression score CES-D < 16	175 (53 · 2 %)	50 (62 · 5 %)	52 (54 · 7 %)	73 (47 · 4 %)
Depression score CES-D > =16	154 (46 · 8 %)	30 (37 · 5 %)	43 (45 · 3 %)	81 (52 · 6 %)
Drug Abuse				
No reported drug use	238 (73 · 5 %)	66 (84 · 6 %)	67 (72 %)	105 (68 · 6 %)
Reported drug use	86 (26 · 54 %)	12 (15 · 38 %)	26 (28 %)	48 (31 · 4 %)
CD4+ T lymphocytes	462 (300)	481 (280)	427 (230)	473 (340)
Therapy				
No therapy	133 (40 · 4 %)	25 (31 · 3 %)	44 (46 · 3 %)	64 (41 · 6 %)
ART	143 (43 · 5 %)	28 (35 %)	40 (42 · 11 %)	75 (48 · 7 %)
cART	53 (16 · 1 %)	27 (33 · 8 %)	11 (11 · 58 %)	15 (9 · 7 %)
Antiretroviral Adherence				
< 95 % therapy use	284 (86 · 3 %)	57 (71 · 3 %)	87 (91 · 58 %)	140 (90 · 9 %)
> = 95 % therapy use	45 (13 · 7 %)	23 (28 · 75 %)	8 (8 · 42 %)	14 (9 · 1 %)
HIV Viral Load	3 · 69 (1 · 18)	3 · 04 (1 · 27)	3 · 86 (1 · 05)	3 · 91 (1 · 09)
Alcohol use > = 7 drinks/week	2 (0 · 63)	14 (4 · 43)	26 (8 · 23)	42 (13 · 29)

*Abbreviations*: *CES-D* Center for Epidemiologic Studies of Depression Scale

Using HIV RNA level set at the level below assay detection as the outcome in the univariate multinomial regression analyses, the constants of being African American and having a lower education, and the time-varying variables including reporting of depressive symptoms, illicit drug use, lower CD4+ T lymphocyte count, unstable housing, moderate or heavy alcohol use and poorer adherence significantly increased likelihood of membership in sustained viremia HIV treatment career (Table 2). Both ART and highly active combination ART (cART) decreased the odds of belonging in a sustained viremia HIV treatment career. Using HIV RNA level set at the level below assay detection as the outcome in the multivariate multinomial regression analysis being African American, low CD4+ T lymphocyte count, not being on ART, alcohol use defined as > = 7 reported drinks per week since last visit and low adherence remained significant predictors of sustained viremia HIV treatment career (Table 2). A sensitivity analysis was performed using an HIV RNA cutoff of >1000 copies/mL to identify group trajectories, and revealed similar distribution of individuals within the three HIV treatment career groups; and univariate analysis revealed a statistically significant increase in membership in viremia career based on African American race, lower education level, drug abuse, and lower CD4+ T lymphocyte count (data not shown). From the multivariate analysis, lower education, African American race, lower CD4+ T lymphocyte count, and no therapy continued to increase significantly risk for membership in sustained viremia HIV treatment careers (data not shown).

**Table 2:** Regression analyses of predictor variables for sustained viremia trajectory (>80 copies/mL or lower limit)

	Univariate analysis		Multivariate analysis	
Variables	*P* value	OR (95 % CI)	*P* value	OR (95 % CI)
Age^a^	0 · 506	1 · 0069 (0 · 9865–1 · 0276)	0 · 0114	1 · 0316 (1 · 0078–1 · 0559)
Education	0 · 006	0 · 5309 (0 · 3360–0 · 8388)	0 · 0309	0 · 586 (0 · 3591–0 · 9562)
Race	0 · 0001	--	--	--
African American	0 · 0082	2 · 1658 (1 · 2214–3 · 8404)	0 · 0153	2 · 1852 (1 · 162–4 · 1093)
Hispanic ethnicity	0 · 0799	0 · 446 (1 · 807–1 · 1011)	0 · 321	0 · 6088 (0 · 2284–1 · 6222)
Other ethnicity	0 · 7244	1 · 2547 (0 · 3554–4 · 4292)	0 · 4003	1 · 7358
(0 · 4802–6 · 274)				
CD4/100^a^	<0 · 0001	0 · 8540 (0 · 8004–0 · 9112)	<0 · 0001	0 · 8743 (0 · 8222–0 · 9297)
Stable housing^a^	0 · 0466	0 · 7068 (0 · 4982–1 · 0027)	0 · 4938	0 · 8879 (0 · 6254–1 · 2606)
Drug abuse^a^	0 · 0006	1 · 8976 (1 · 2787–2 · 8159)	0 · 3029	1 · 2341 (0 · 8096–1 · 8812)
Depression^a^	<0 · 0001	1 · 8559 (1 · 3826–2 · 4912)	0 · 0413	1 · 3608
(1 · 019–1 · 8172)				
Adherence (> = 95 %)^a^	<0 · 0001	0 · 3282 (0 · 2596–0 · 4148)	0 · 0049	0 · 6804 (0 · 5206–0 · 8892)
Antiretroviral Therapy^a^	<0 · 0001	--	<0 · 0001	--
ART^b^	0 · 0007	0 · 4858 (0 · 3197–0 · 7381)	<0 · 0001	0 · 3878 (0 · 2486–0 · 6048)
cART	0 · 0001	0 · 2488 (0 · 1711–0 · 3616)	<0 · 0001	0 · 2851 (0 · 1833–0 · 4434)
Alcohol use > = 7 drinks/week since last visit^a^	<0 · 0001	2 · 4786 (1 · 5721–3 · 908)	0 · 0352	1 · 5921 (0 · 9956–2 · 546)

Referents were less than high school for education, non-Hispanic caucasian for race, no private housing since last visit for housing, no reported drug use since last visit for drug abuse, <16 CESD for depression, adherence <95 % for adherence, and no antiretroviral drug use for therapy

*Abbreviations*: *ART* antiretroviral therapy, *CI* confidence interval, *CES-D* Center for Epidemiologic studies of depression scale, *cART* highly active combination antiretroviral therapy, *HIV* human immunodeficiency virus, *OR* odds ratio

^a^time-varying variable

^b^Survey measures differently from highly active antiretroviral therapy

**Fig. 2:**
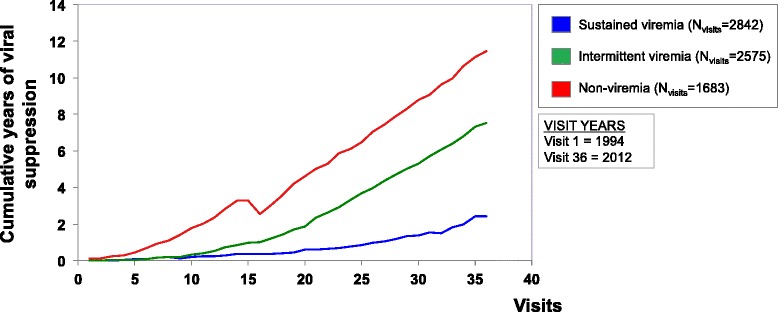
Cumulative viral suppression-years as the outcome of analysis using HIV RNA detection level >80 copies/mL show three distinct HIV treatment careers

Analysis using cumulative viral suppression-years as the outcome similarly identified three distinct HIV treatment careers (Fig. 2). Non-viremia HIV treatment careers experienced the highest numbers of years of cumulative viral suppression. Intermittent viremia HIV treatment careers saw intermediate numbers of cumulative viral suppression-years, while sustained viremia HIV treatment careers observed the lowest numbers of cumulative viral suppression-years.

**Fig. 3:**
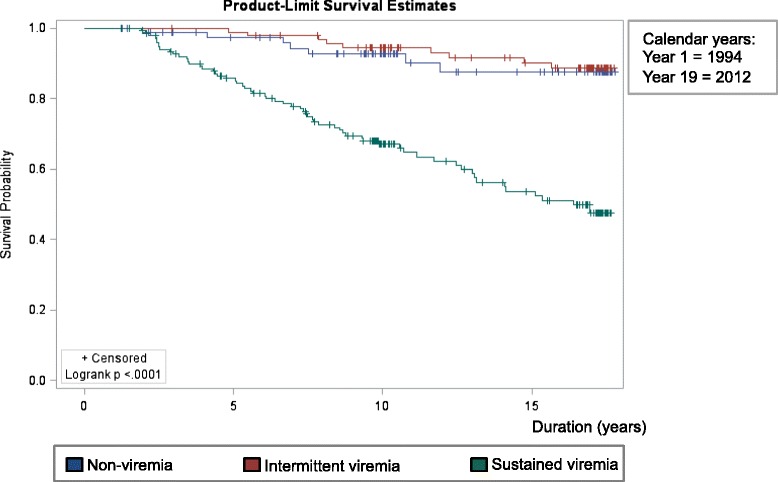
Kaplan-Meier survival analysis for participants (*N* = 329) showed a significant difference between sustained viremia (*N* = 154) survival trends from intermittent viremia (*N* = 95) and non-viremia (*N* = 80) HIV treatment careers using >80 copies/mL HIV RNA or lower at time of assay detection limit with (+) signs indicating censored observations and survival time defined as age at last visit seen alive minus age at first visit

Overall mortality rates differed significantly based on HIV treatment careers. The highest mortality rate was 31.1 % in sustained viremia HIV treatment careers, 6.9 % in intermittent viremia HIV treatment careers, and 4.9 % in non-viremia HIV treatment careers. Kaplan-Meier analysis revealed a significant effect of HIV treatment career on survival probability (Fig. 3). Survival curves for non-viremia and sustained viremia HIV treatment careers varied significantly (*P* < 0.0001) whereas non-viremia and intermittent viremia HIV treatment careers did not (*P* = 0.7647) (Fig. 3). Univariate analyses from Cox proportional hazard model showed that constants of being of African American race and older age at first visit in study, and time-varying covariates including having depressive symptoms, drug abuse, lower CD4+ T lymphocyte count, and belonging to the sustained viremia HIV treatment career increased mortality hazard (Table 3). From the multivariate analysis, older age at first visit in study, lower CD4+ T lymphocyte count, and belonging to the sustained viremia HIV treatment career remained significant risks for premature all-cause mortality (Table 3). Although alcohol use was associated with sustained viremia in multinomial regression analyses, Cox analyses showed it was not associated with mortality.

**Table 3:** Univariate and multivariate Cox proportional hazard models: the association of treatment careers and co-factors with mortality in the DC WIHS (*n* = 329)

Variables	Univariate analysis		*P* value	Multivariate analysis		*P* value
	HR	(95 % CI)		HR	(95 % CI)	
Race			0 · 09			0 · 94
White	Referent					
Black	2 · 49	(1 · 08,5 · 76)	0 · 03	1 · 2	(0 · 47,3 · 07)	0 · 71
Hispanic	1 · 02	(0 · 26,4 · 09)	0 · 98	0 · 81	(0 · 15,4 · 31)	0 · 8
Other	1 · 88	(0 · 38,9 · 35)	0 · 44	1 · 25	(0 · 14,11 · 13)	0 · 84
Education						
< 12 years	Referent					
> = 12 years	0 · 75	(0 · 46,1 · 24)	0 · 26			
Age	1 · 05	(1 · 03,1 · 08)	0 · 0003	1 · 06	(1 · 02,1 · 09)	0 · 004
Housing^a^						
No housing	Referent					
Housing	0 · 86	(0 · 48,1 · 53)	0 · 6			
Depression^a^						
Depression score <16	Referent					
Depression score > =16	1 · 71	(1 · 02,2 · 86)	0 · 04	0 · 94	(0 · 54,1 · 64)	0 · 83
Drug Abuse^a^						
No reported drug use	Referent					
Reported drug use	2 · 01	(1 · 2,3 · 37)	0 · 008	1 · 57	(0 · 91,2 · 7)	0 · 11
CD4^a^	0 · 65	(0 · 56,0 · 75)	<0 · 0001	0 · 69	(0 · 6,0 · 8)	<·0001
ART^a^						
No ART use	Referent					
ART use	1 · 69	(0 · 97,2 · 93)	0 · 06	1 · 09	(0 · 58,2 · 04)	0 · 8
cART^a^						
No cART use	Referent					
cART use	1 · 06	(0 · 62,1 · 8)	0 · 84			
Adherence^a^						
< 95 % therapy use	Referent					
> = 95 % therapy use	0 · 64	(0 · 32,1 · 27)	0 · 2			
HIV treatment career			<0 · 0001			<·0001
Nonviremia	Referent					
Intermittent	0 · 83	(0 · 31,2 · 24)	0 · 71	0 · 59	(0 · 18,1 · 94)	0 · 38
Viremia	5 · 25	(2 · 4,11 · 47)	<0 · 0001	3 · 967	(1 · 53,10 · 28)	0 · 0045
Alcohol use > = 7 drinks/week since last visit^a^	1 · 432	(0 · 704,2 · 912)	0 · 322			

Multivariate model included race, age, depression, drug abuse, CD4, ART and HIV treatment career

*Abbreviations*: *CI* 95 % confident interval, *CES-D* Center for Epidemiologic studies of depression scale, *cART* highly active antiretroviral therapy, *HR* Hazard ratio

^a^: time-dependent variables

## Discussion

### Novel approach to understanding long-term HIV care

**Fig. 4:**
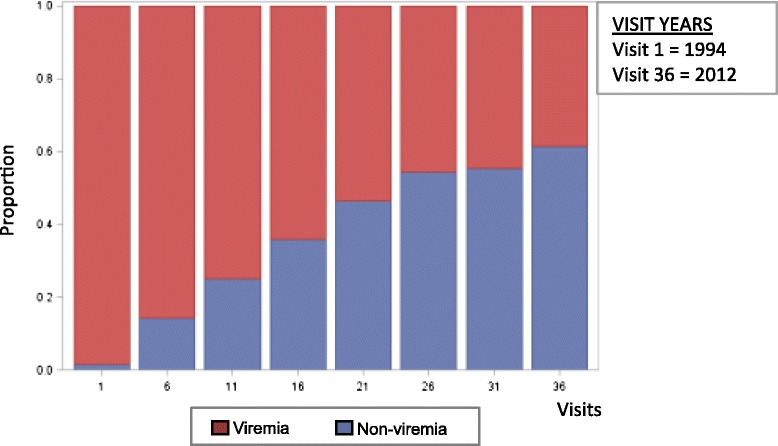
Cross-sectional depiction of the proportion of DC WIHS women with viremia and women without virema 1994–2012 (*N* = 266)

This analysis using group-based probability trajectories provides a unique perspective of the dynamic nature of HIV treatment over several decades. Data from this long-term observational cohort study in a high HIV-prevalence community identified three distinct, but generalizable trajectories among individuals living with HIV. The population as characterized by virologic responses over decades included distinct long-term treatment “careers” defined as individuals with low (non-viremia), moderate (intermittent viremia), and high (sustained viremia) based on the probability of detectable HIV RNA over time. These longitudinal patterns were not observable in more static analyses such as calculated proportions of viremia and non-viremia in the same cohort (Fig. 4). More specifically, this group-based probabilistic trajectory analysis identified a significantly large pool of individuals with intermittent viral suppression, a phenomenon that is not captured in the current HIV care continuum. These findings therefore challenge more traditional serial cross-sectional analyses of the HIV care continuum in DC using clinic-based viral data and reporting viral suppression rates from 57.4 to 61 % among HIV-infected individuals [24, 25]. We verified our observations using a separate analysis of cumulative years of viral load suppression, which are related to, but not synonymous with other viral load-based analyses, including cumulative viral load, and community viral load [26, 27].

In this study among women representative of the community, there is an overall increase over time in the proportion of individuals who achieve viral suppression. Similar results have been demonstrated in a study from British Columbia, with a gradual decline in individuals with viremia over the course of decades [28].

Our approach is unique both in the population sampling strategy, as well as the analytic approach, the results of which capture the complexity of HIV care in the context of a modern, urban setting. HIV-positive women within the DC WIHS receive HIV treatment in an array of medical settings, including approximately 40 private practitioners, eight community-based clinics, six regional hospitals, and five academic centers. However, engagement in care is not a pre-requisite to study enrollment and follow up, and given the community outreach-based recruitment of participants this cohort provides a unique non-clinic based measurement of participation in HIV treatment. Our findings that less than one third of women achieve and sustain viral suppression is particularly noteworthy given that this is a research-primed cohort with access to HIV-outreach in the course of their study participation. Since HIV treatment is not provided as part of this study, this ecologic setting reflects the changing efficacy of outreach, management and care among women.

Our study contributes to the growing literature in this important area of research examining the more chronic nature of living with HIV, and is unique in the ability to demonstrate the relationship between intermittent viral suppression and long-term survival. Current clinical guidance is driven by the benchmarks that were established for the rapid approval of antiretroviral medications for treatment of HIV in the early days of the HIV epidemic. We were not able to demonstrate a significant difference in survival between individuals with sustained viral suppression and intermittent viremia in this cohort with long-term follow up. This finding is surprising given the findings in the SMART study that demonstrated an increase in overall morbidity and mortality associated with intermittent (though structured) treatment interruption [29]. Our study population differs significantly from the SMART study population, however, suggesting that the SMART study findings may not be generalizable to an all female, younger primarily African American cohort. Notable in our findings is the lack of association between cART and survival. The identified treatment trajectories and viremia is reflective of effective cART use, and modeling of survival by trajectory groups results in co-linearity and non-significant association of cART on survival. Interpretation of that finding should thus be tempered, as viremia outcomes are strongly associated with cART use and adherence as shown in table 2.

### Limitations and future directions

Our analytic approach was able to capitalize on the rich longitudinal data available on the women included in this study. The identified group trajectories are suggestive of treatment successes and failures within this region, but are not necessarily generalizable to other sub-populations within the region or throughout the United States. Further studies are needed to determine applicability to other regions. While beyond the scope of this initial study, future studies should include qualitative methods to identify potential interventions that could be enhance individualized treatment outcomes over time. Finally, HIV treatment guidelines and available treatment regimens have evolved since the early days of the HIV epidemic, and the visualized inflection points within the identified treatment careers may represent improvement in available antiretrovirals over the course of the decades, culminating in the availability of various fixed dose single tablet ART formulations. This is reflected in the declining near convergence in probability of viremia among the intermittent viremia, and non-viremia treatment career arms demonstrated here (Fig. 1). Additionally, individuals with intermittent viremia may be at highest risk of transmitting resistant virus. Further characterizing these distinct HIV treatment careers is imperative to improve health outcomes and decrease the potential for ongoing HIV transmission.

The persistence of higher probability of the sustained viremia treatment career despite the availability of better tolerated formulations within the recent years reinforces the continued presence of a group that is particularly difficult to engage in care, a sub-group at higher risk of mortality for whom defined strategies for engagement in care are lacking. Our findings highlight the importance of psychosocial factors in the ability of women to achieve and maintain viral suppression including the effect of marginal housing and moderate or greater alcohol use. These data corroborate findings from other studies, and underscore the importance of adequately addressing treatment resilience to maintain long-term viral suppression [30–32].

## Conclusions

Women of color are disproportionately affected by the HIV epidemic in the United States, and the identified HIV treatment careers in this study are likely highly representative for this important population. Further research is needed to determine generalizability of our findings to other populations. More detailed information about the risk and protection factors of HIV should be examined within the framework of HIV treatment careers, including the relationship between HIV treatment careers, the development of co-morbidities, and overall quality of life among DC WIHS participants. Moreover, life course interviews and focus groups discussions could provide further insight into the risk and protection factors that contribute to longitudinal HIV treatment outcomes. Such additional studies could identify potentially modifiable structural, biographical, and clinical risk factors to guide effective HIV treatment programs and policy.
